# Is blood eosinophilia a treatable trait in chronic obstructive airway diseases?

**DOI:** 10.3389/fimmu.2026.1868910

**Published:** 2026-06-01

**Authors:** Giulio Massetti, Manuel Martella, Cristina Ruini, Valentina Ruggieri, Bianca Beghe

**Affiliations:** 1 Department of Medical and Surgical Sciences, Section of Respiratory Diseases, University of Modena and Reggio Emilia, Modena, Italy; 2 Department of Life Sciences, University of Modena and Reggio Emilia, Modena, Italy

**Keywords:** asthma, biologics, COPD, endotype, interleukin-5 (IL-5), T2 inflammation

## Abstract

Nowadays, the management of chronic obstructive airway diseases is shifting from traditional diagnostic labels toward a precision medicine approach based on “treatable traits.” Among these, blood eosinophilia is emerging as a biomarker of type 2 (T2) inflammation across asthma and chronic obstructive airway diseases (COPD), even if its role as diagnostic tool and therapeutic target remains debated. In this manuscript we presented a clinical case of a 60-year-old male, former mild smoker (5 p/y), initially diagnosed with severe COPD with persistent airflow limitation, frequent severe exacerbations and persistent eosinophilia, but a history of asthma and nasal polyposis. After six months with optimization of inhaled therapy and clinical reassessment, the diagnosis of “severe COPD” shifted to the diagnosis of “severe eosinophilic asthma”. Considering the new diagnosis, he was prescribed mepolizumab on top of triple inhaled therapy. After 1 year, the clinical response was excellent in terms of symptoms control, exacerbations reduction and OCS sparing, despite no improvement in lung function. Starting from this clinical case, the aim of this mini review is to describe the role of eosinophilic inflammation in asthma and COPD. While eosinophilic inflammation is a common feature of asthma, COPD is usually characterized by neutrophilic inflammation, even if, a subset of patients with COPD has eosinophilic inflammation. In this context, blood eosinophilia might represent a clinically actionable trait to guide diagnosis and personalized therapy in chronic airway diseases even if it should not be targeted in isolation but be integrated into a multidimensional assessment.

## Introduction

Over the last decade, the clinical management of obstructive airway diseases is undergoing a substantial conceptual shift, moving away from rigid nosological classifications —namely asthma, chronic obstructive pulmonary disease (COPD), and asthma–COPD overlap (ACO) (1–3)—towards a personalized, trait-based approach known as the “Treatable Traits” model. This framework emphasizes the identification of specific, clinically relevant traits underpinned by measurable biomarkers and inflammatory endotypes, with the aim of guiding targeted therapy beyond traditional diagnostic labels (4–7).

In this context, blood eosinophilia has emerged as a practical, inexpensive, and reproducible biomarker for the identification of type 2 (T2) inflammation. Although historically considered a hallmark of asthma, T2 inflammation is now recognized in a consistent proportion of patients with COPD, with prevalence estimated ranging from 20% to 40% (8–11). Presenting a clinical case of a man with persistent blood eosinophilia, but uncertain diagnosis of asthma and/or COPD, the aim of this article is to report the role of eosinophilic inflammation in asthma and COPD and to discuss the “persistent eosinophilia” as treatable trait for the management of chronic obstructive diseases.

## Case report

**Figure 1:**
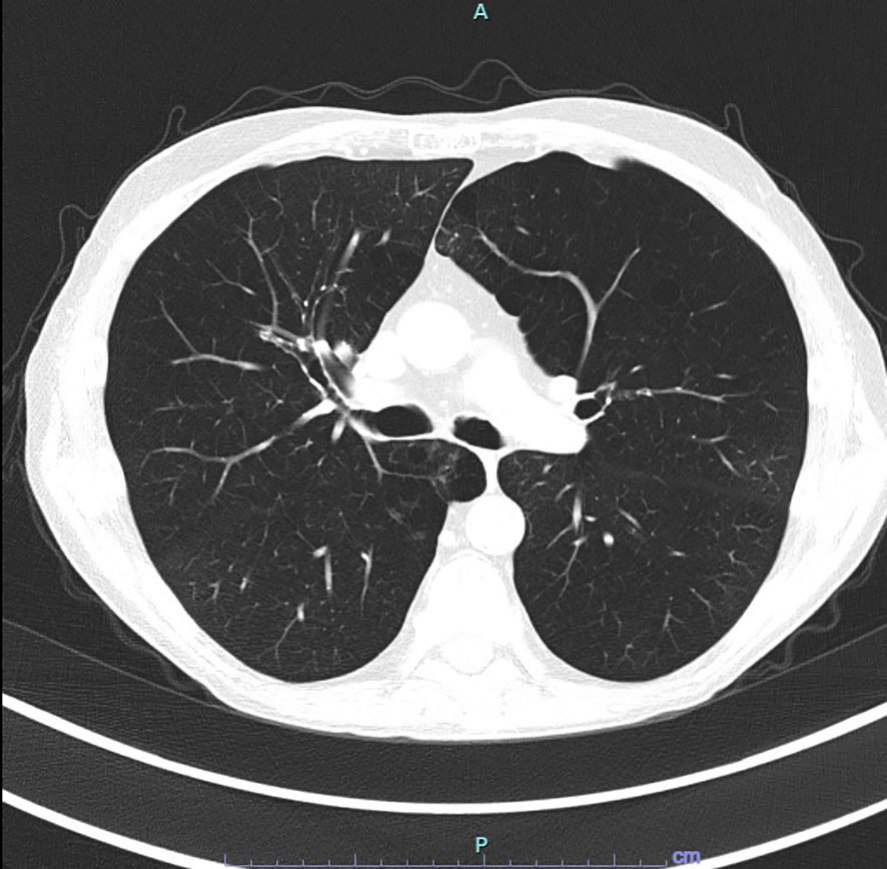
High resolution chest CT scan at baseline showing panlobular emphysema.

**Figure 2:**
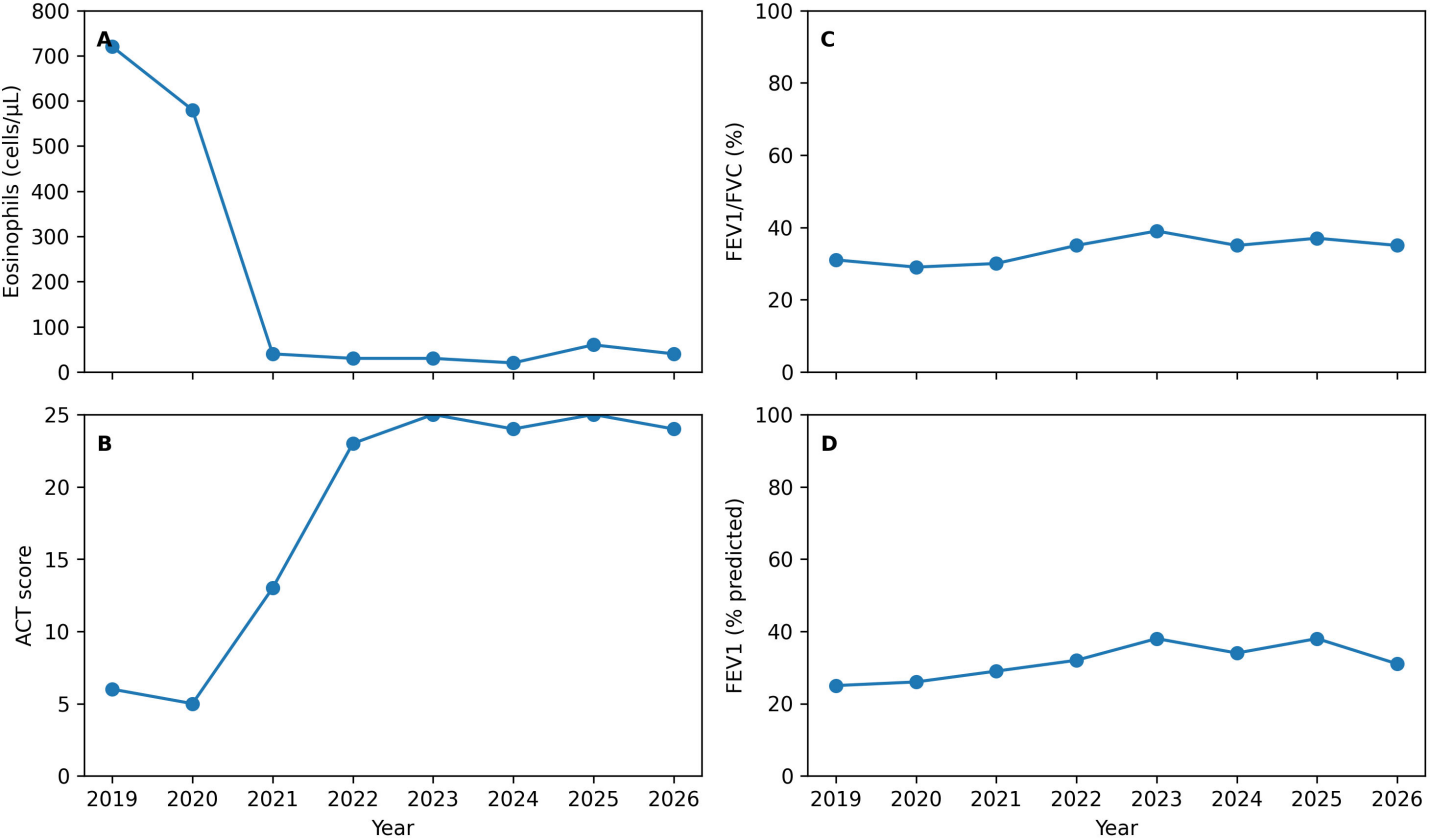
Longitudinal assessment of clinical and functional parameters. **(A)** blood eosinophil count (cells/µL); **(B)** ACT, Asthma Control Test; **(C)** FEV1/FVC %, Forced Expiratory Volume 1 second/Forced Vital Capacity. **(D)** FEV1, Forced Expiratory Volume 1 second (% predicted).

We report a case of a 60-year-old male, retired bank employee, former light smoker (5 p/y), referred nearly 6 years ago to our outpatient clinic with a diagnosis of severe COPD established 3 years earlier. The patient presented with severe fixed airflow obstruction (post bronchodilator FEV1/FVC 28, FEV1 24% pred.) and a history of frequent moderate-to-severe exacerbations (> 3 per year) despite full adherence to maximal triple inhaled therapy (ICS/LABA/LAMA). High-resolution computed chest tomography revealed diffuse panlobular emphysema (**Figure 1**), while serum alpha-1 antitrypsin levels were within normal limits (127 mg/dl). Nonetheless, the overall clinical phenotype appeared atypical for smoking-related COPD. A pivotal finding in the diagnostic reassessment was the presence of persistent blood eosinophilia (mean blood eosinophils count (BEC) >700 cells/μL documented across multiple evaluations while total IgE level was 117 IU/ml). Furthermore, the patient’s reported history of asthma (never formally diagnosed and properly treated) and of nasal polyposis requiring surgical intervention, suggested a T2 inflammatory endotype. These findings raised a critical diagnostic question: whether the patient should be classified as having COPD with eosinophilic inflammation or, alternatively, severe eosinophilic asthma (SEA) with fixed airflow limitation resulting from long-standing disease with a mild smoking exposure. Integration of functional parameters—in particular peak expiratory flow (PEF) variability exceeding 10%, together with the persistence of blood eosinophilia, suggested a revised diagnosis of SEA. Hence, therapeutic focus shifted from irreversible emphysematous damage to the modifiable IL-5–driven inflammatory pathway. Mepolizumab was initiated as add-on biologic therapy. After 12 months, the patient exhibited a marked clinical improvement, characterized by enhanced symptom control (ACT: 24), a substantial reduction in exacerbation frequency (more than 3 per year to 1 per year), and a significant steroid-sparing effect, in the absence of significant improvement in lung function (**Figure 2**). These clinical benefits have been stable over a follow-up of six years.

## Blood eosinophilia in chronic obstructive airway diseases

Eosinophils are terminally differentiated granulocytes derived from CD34+ hematopoietic progenitor cells in the bone marrow (3, 12–14). Their differentiation, maturation, release into the circulation, and survival in peripheral tissues are critically regulated by interleukin-5 (IL-5), which represents the key cytokine governing eosinophil homeostasis. Due to its biological pleiotropic effects (15, 16), IL-5 not only promotes terminal differentiation and bone marrow egress, but also inhibits eosinophil apoptosis in target tissues. Recent evidence highlights that eosinophils operate within a complex and dynamic immune network rather than as isolated effector cells. They interact closely with Th2 lymphocytes and type 2 innate lymphoid cells (ILC2s), the principal cellular sources of IL-5, IL-4, and IL-13 (17, 18). In addition, eosinophils contribute to T2 inflammation through the release of cytokines, chemokines, and lipid mediators, reinforcing a bidirectional amplification loop within the airway microenvironment (19). At the tissue level, eosinophilic inflammation is sustained through bidirectional interactions with the bronchial epithelium. Environmental insults and endogenous stimuli induce epithelial release of alarmins, including thymic stromal lymphopoietin (TSLP), interleukin-33 (IL-33), and interleukin-25 (IL-25), which act as upstream regulators of type 2 immunity and activate multiple immune cell subsets, including ILC2s, Th2 cells, and eosinophils (19). Following recruitment to the airways, activated eosinophils undergo degranulation, releasing cytotoxic mediators including major basic protein (MBP) and eosinophil peroxidase (EPO) (20). These proteins contribute to epithelial injury, disruption of tight junctions, airway remodeling, and bronchial hyperresponsiveness. In addition, reciprocal interactions with mast cells further potentiate broncho-constrictive mediator release, reinforcing a self-perpetuating inflammatory environment (21).

Despite the strong biological rationale supporting their clinical relevance, the use of BEC as a therapeutic guide has historically been questioned because of temporal variability. Moreover, BEC may be acutely influenced by several clinical factors commonly encountered in daily practice. In particular, maintenance oral corticosteroids (OCS) or recent exacerbations treated with systemic corticosteroids may transiently suppress eosinophil levels, potentially masking an underlying T2 inflammatory endotype and reducing the reliability of a single measurement during the diagnostic assessment (2, 10, 22). In fact, whenever possible, BEC should be interpreted longitudinally and in the context of clinical stability, avoiding measurements obtained shortly after systemic corticosteroid exposure. Interestingly, recent data from longitudinal studies, including the analyses by Baraldi et al. (23), report that although intra-individual variability exists, persistently elevated BEC represent a robust and clinically meaningful signal.

Systematic reviews and meta-analyses have consistently demonstrated that elevated blood eosinophil levels are associated with an increased risk of exacerbations both in asthma and COPD (12, 13, 24) and, importantly, predict responsiveness to inhaled corticosteroids (ICS) (2, 25–28) and to biologics targeting the IL-5 pathway (29). Taken together, these findings support that blood eosinophilia should be regarded not merely as a biomarker of inflammation, but as a clinically actionable treatable trait. However, as recently reported by Lommatzsch et al, eosinophilia has different impact across distinct asthma phenotypes and should not be targeted as an independent therapeutic goal but rather used as an additional diagnostic tool for an accurate diagnosis (30).

### Blood eosinophilia in asthma

Eosinophilic airway inflammation is the hallmark of asthma. As extensively discussed by Papi et al. (31), the identification of eosinophilia allows for the deconstruction of the clinical complexity of asthma, enabling a precision medicine approach that targets the underlying T2 inflammatory drive. This biological trait is not merely a marker of disease presence, but serves as a primary driver of airway remodeling and hyperresponsiveness. The clinical utility of monitoring eosinophilic inflammation is further underscored by Porsbjerg et al. (22), who highlighted the critical role of biomarkers such as BEC and fractional exhaled nitric oxide (FeNO) in predicting clinical trajectories: indeed, the presence of persistent eosinophilia, even in patients treated with ICS, identifies a high-risk population prone to frequent and severe exacerbations.

A crucial aspect of managing this trait in asthma is its longitudinal variability even if evidence indicates that BEC levels remain relatively consistent over time, with a high proportion of asthmatic patients staying above the clinically relevant threshold of 300 cells/μL (32). This persistence is a strong predictor of “type 2-high” activity and identifies individuals at risk for progressive lung function decline (8). Furthermore, the stability of this biomarker is essential for the reliable selection of patients for biologic treatments (33).

### Blood eosinophilia in COPD

Although COPD has traditionally been regarded as a neutrophilic inflammatory disease, recent observations suggest that 20–30% of patients exhibit eosinophilic inflammation (13). A persistent eosinophilic phenotype in COPD carries significant prognostic implications, though the reliability of eosinophils as a predictive biomarker is still debated. In a large-scale *post-hoc* analysis of 11 clinical trials involving 22,125 patients, Singh et al. found that 45.6% of the population had a baseline BEC > 2% (defined in their study as >150 cells/μL). However, the authors concluded that there was no clinically significant relationship between baseline BEC and exacerbation rates, suggesting that a patient’s previous exacerbation history is a far more reliable predictor of future events. In contrast, the meta-analysis by Chen et al. (12), including 21 studies and nearly 80,000 patients, supported the association between eosinophilic inflammation and prognosis. Their findings suggest that high BEC (defined as >300 cells/µL or >2%) are significantly associated with a higher annual rate of moderate-severe exacerbations, compared to the paucigranulocytic endotype (12). Indeed, eosinophils may act not only as bystanders of inflammation but as key pathogenic driver. The release of cytotoxic granular proteins, such as MBP, may contribute to airway hyperresponsiveness, tight junction disruption, and subsequent mucosal injury (20). The adoption of blood eosinophilia as a routine biomarker in COPD patients has historically been limited by concerns regarding intra-individual variability. However, recent longitudinal studies provided a clearer framework for the stability of this trait. Cohort studies have shown that approximately 70% of COPD patients maintain their eosinophil category—according to GOLD thresholds (<100, 100–300, and ≥300 cells/µL)—over one year of follow-up (29, 32, 34–36). In particular, Long et al. (32) reported strong statistical reliability for this biomarker, highlighting an intraclass correlation coefficient (ICC) of 0.84 and a Spearman’s rho of 0.71. Their data also showed that COPD patients with the lowest counts exhibited the greatest stability, as 85.3% of subjects with baseline eosinophils <100 cells/µL maintained stable counts after one year, even if more recent evidence by Baraldi et al. (24) indicate that approximately 40–44% of newly diagnosed patients may experience eosinophils count fluctuations.

From a therapeutic prospective, BEC emerged as the most accurate predictor of clinical response to ICS for exacerbation prevention (2). As described by Brightling and Greening (4), the relationship between eosinophil levels and ICS efficacy follows a log-linear dose–response pattern: patients with counts <100 cells/µL derive minimal or no benefit from ICS and are primarily exposed to pneumonia risk. Conversely, in individuals with counts >300 cells/µL, ICS intensification is strongly recommended by international guidelines, as it is crucial to suppress airway inflammation and drastically mitigate clinical instability (28, 37). Finally, eosinophilic inflammation in COPD is emerging as a target for biologics targeting T2 inflammation (2).

### Blood eosinophilia in ACO

In older patients, the clinical features of asthma and COPD often converge into a phenotype known as ‘Asthma–COPD Overlap’ (ACO), recently neglected as a disease entity (1, 2). However, this condition is associated with a significantly more severe clinical course, including an accelerated lung function decline and increase risk of exacerbation than in COPD alone (38–40). According to Takayama et al. (41), BEC is a key diagnostic marker for “ACO”, with a cut-off of >150 cells/µL being highly indicative of this entity. Notably, their findings also highlight that patients with ACO who present with higher BEC and elevated FeNO levels are at increased risk for severe exacerbations, necessitating more intensive monitoring and targeted anti-inflammatory therapy.

## Biological therapies in chronic obstructive diseases

While biologics for severe asthma have been available for about 20 years, therapeutic landscape for COPD has historically been dominated by inhaled bronchodilators and corticosteroids in patients with frequent exacerbations. However, the recognition of blood eosinophilia as a “treatable trait” has paved the way for the integration of monoclonal antibodies into clinical practice even for patients with COPD (42). Biologics specifically targeting the T2 inflammation might be effective in the subset of COPD patients with eosinophilic endotype and history of frequent exacerbations (8, 23, 24).

### Anti-interleukin 5 monoclonal antibodies: mepolizumab and benralizumab

Mepolizumab, a humanized monoclonal antibody that targets interleukin-5, was the first biologic extensively studied in SEA and more recently in severe eosinophilic COPD. The clinical development of mepolizumab underscores the need of biomarker-driven patient selection in the management of severe asthma. In early clinical investigations, mepolizumab significantly reduced blood eosinophil levels in an unselected population of severe asthmatic patients without achieving the expected clinical results (43). In contrast, when it was administered in patients with SEA, the reduction of blood and sputum eosinophilia was associated with a significant decrease in asthma exacerbations and improvement of lung function (44, 45). The following clinical trials (46–48) and real-world studies (49–51) showed decrease of severe exacerbations by 50%, improvement in quality of life and reduction in OCS use. In addition, long-term safety and durability of response was finally validated by the extension COLUMBA study (52).

The use of mepolizumab in COPD hinges on the hypothesis that a subset of patients exhibits a predominant T2 inflammatory endotype, characterized by persistent airway and systemic eosinophilia. Unlike the robust and consistent outcomes observed in SEA, the clinical efficacy of IL-5 inhibition in COPD has yielded more nuanced results (53). The primary evidence stems from two phase III randomized controlled trials, METREX and METREO (54). In the METREX study, mepolizumab (100 mg subcutaneous) showed a significant reduction in the annual rate of moderate-to-severe exacerbations compared to placebo, specifically in severe COPD patients with an eosinophilic phenotype (defined as BEC ≥150 cells/μL at screening or ≥300 cells/μL in the previous year), whereas a significant reduction of exacerbations was not reached in the METREO study, designed to evaluate the efficacy of higher doses of mepolizumab (100 mg and 300 mg). However, a *post-hoc* analysis of both trials suggested a clear trend toward benefit as blood eosinophil counts increased (54). More recently, the MATINEE study (55) showed that, among patients with severe COPD with a history of exacerbations and an eosinophilic phenotype (≥300 cells/µL), mepolizumab significantly reduced the annualized rate of moderate and severe exacerbations when added to triple inhaled therapy, underscoring the crucial role of appropriate patient selection, as well as the need for both phenotyping (e.g., exacerbation history) and endotyping (e.g., blood eosinophilia) (28, 56). Finally, in a recent real-world study, mepolizumab significantly reduced blood eosinophil counts and exacerbation rates in patients with ACO, supporting the pathogenic role of eosinophilia in this population (53).

Benralizumab is a monoclonal antibody that binds to the IL-5 receptor alpha chain (IL-5Rα) leading to a near-complete depletion of eosinophils in blood and target tissues via antibody-dependent cell-mediated cytotoxicity (ADCC). In SEA (defined by baseline BEC ≥300 cells/μL), the phase III trials SIROCCO and CALIMA established its efficacy, showing reduction in the annualized rate of exacerbations up to 51%, and a rapid improvement in lung function (57, 58). Following trials further confirmed the efficacy showing a potent OCS sparing effect, and these findings have been supported by several real-world evidence studies also in patients with different comorbidities, in particular nasal polyps (59–61).

In COPD, the clinical trajectory of benralizumab is more challenging. The phase III GALATHEA and TERRANOVA trials investigated the efficacy of benralizumab in patients with moderate-to-very-severe COPD and a history of frequent exacerbations. Despite the clear identification of an eosinophilic trait (BEC was 453.2 ± 280.25 cells/μL for GALATHEA and 504.5 ± 393.08 cells/μL for TERRANOVA in the primary analysis population), both trials failed to reach the primary endpoint of a statistically significant reduction in exacerbations (62).

The different response to anti-eosinophil monoclonal antibodies in asthma and COPD likely reflects the distinct biological role of eosinophils in the two diseases. In SEA, eosinophils represent a central effector of T2-driven inflammation and directly contribute to airway hyperresponsiveness, mucus production, and structural remodeling. Consequently, IL-5 pathway inhibition may profoundly suppress a key pathogenic mechanism. In contrast, COPD is characterized by a far more heterogeneous inflammatory milieu, involving neutrophilic inflammation, macrophage activation, cigarette smoke-induced innate immune dysfunction, emphysematous destruction, mucus hypersecretion, and recurrent infections (4, 8, 10). In this setting, blood eosinophilia may identify a subgroup with partial T2 inflammatory activation, but eosinophils are unlikely to represent the dominant driver of disease in the majority of patients. This pathophysiological complexity might explain the less consistent clinical efficacy of eosinophil-depleting therapies observed in COPD compared with asthma.

### IL-4 and 13 pathways inhibition: dupilumab

Dupilumab, by targeting the IL-4Rα subunit, effectively modulates the signaling of both IL-4 and IL-13, which are key drivers of goblet cell hyperplasia, IgE synthesis, airway hyperresponsiveness and mucus production. In this context, increasing evidence highlights the relevance of mucus plugs as a pathological hallmark of T2–driven airway disease. Mucus plugs, resulting from excessive mucin production (particularly MUC5AC) and impaired mucociliary clearance, have been associated with airflow obstruction, ventilation heterogeneity, and exacerbation risk in both asthma and COPD (63, 64). IL-13 plays a central role in promoting goblet cell metaplasia and mucin gene expression, thereby contributing to the formation of these obstructive plugs. Mucus plugs are increasingly emerging as a potential radiological treatable trait in chronic obstructive airway diseases. Recent imaging studies showed that mucus plugging assessed by chest computed tomography is associated with airflow obstruction, exacerbation risk, eosinophilic inflammation, and longitudinal lung function decline, independently of spirometric values (63, 64). In this context, radiological identification of mucus plugs may help to phenotype patients with predominant IL-13–driven disease and could represent a measurable biomarker to guide biologic therapies. This approach further supports the transition toward precision medicine strategies based on specific biological and structural endotypes rather than conventional diagnostic labels alone. In SEA, the phase III LIBERTY ASTHMA QUEST trial showed that dupilumab significantly reduced severe exacerbation rates by up to 48% and induced rapid and sustained improvements in lung function, with the greatest benefit observed in patients with elevated BEC and FeNO (65). Consistently, the LIBERTY ASTHMA VENTURE trial further demonstrated a significant OCS sparing effect (66). In COPD patients, dupilumab has shown to be successful in reducing exacerbations. In the phase III BOREAS and NOTUS trials conducted in patients with moderate-to-very-severe COPD and evidence of T2 inflammation (BEC > 300 cells/mL), dupilumab significantly reduced the annualized rate of moderate-severe exacerbations by approximately 30% and led to a rapid, significant improvement in FEV1 compared to placebo (67). These landmark results suggest that the dual inhibition of IL-4 and IL-13 may address the heterogeneity of COPD more effectively than targeting eosinophils alone, potentially by mitigating mucus hypersecretion, mucus plug formation and structural remodeling alongside inflammatory control. In this context, dupilumab has become the first biologic to receive regulatory approval for the treatment of a specific T2 inflammatory “treatable trait” in COPD, marking a new era in the personalized management of the disease.

## Conclusions

The clinical case presented in this manuscript highlights the need to move beyond traditional diagnostic labels toward a precision medicine approach based on “treatable traits.” The persistence of blood eosinophilia in the patient with the diagnosis of severe COPD, because of radiological evidence of emphysema and fixed airflow limitation, but limited smoking exposure, self-reported history of asthma and nasal polyps let us to identify a key pathogenic pathway and reevaluate the diagnosis, enabling the introduction of mepolizumab, which at the time was approved only for SEA. Focusing on the specific biological pathway, the persistent eosinophilia, allowed a personalized treatment. This proactive approach, as recently discussed by Porsbjerg et al. (68), might allow to intercept the disease during its ‘florid’ stage and to suppress inflammatory activity with clinical improvements. Unfortunately, it was not timely enough to prevent structural and irreversible remodeling. Persistent eosinophilic inflammation may contribute to fixed airflow obstruction through several mechanisms. Activated eosinophils release different mediators (e.g. cytotoxic granule proteins, transforming growth factor-β (TGF-β), matrix metallo-proteinases, and pro-fibrotic mediators) and promote epithelial injury, sub-epithelial fibrosis, smooth muscle hypertrophy, goblet cell hyperplasia, and extracellular matrix deposition (21, 31, 69, 70) leading to irreversible airway narrowing, even when the inflammatory component is controlled (68–70). This might explain why our patient experienced marked clinical improvement with mepolizumab despite no change has been observed in lung function. In this context, early identification of persistent blood eosinophilia may be clinically relevant not only to predict treatment responsiveness, but also to intercept the disease before structural changes become irreversible. Indeed, increasing evidence suggests that early suppression of T2 inflammation may modify the trajectory of airway remodeling and potentially prevent the transition from active inflammatory disease to fixed airway damage (21, 31, 68, 70, 71). From this view, blood eosinophilia could represent a key marker for an early interception of the disease. Nevertheless, important gaps remain regarding the relationship between circulating eosinophils and tissue eosinophilic inflammation, as blood eosinophilia does not always accurately reflect airway cellular infiltration or local inflammatory activity (10, 29). Future research aimed at better defining the correlation between blood and tissue eosinophilia, integrating molecular, functional, and radiological biomarkers, will be essential to further refine the treatable traits model and improve precision medicine strategies in chronic obstructive airway diseases.
